# Resource supply and the evolution of public-goods cooperation in bacteria

**DOI:** 10.1186/1741-7007-6-20

**Published:** 2008-05-14

**Authors:** Michael A Brockhurst, Angus Buckling, Dan Racey, Andy Gardner

**Affiliations:** 1 School of Biological Sciences, Biosciences Building, University of Liverpool, Crown Street, Liverpool, L69 7ZB, UK; 2 Department of Zoology, University of Oxford, South Parks Road, Oxford, OX1 3PS, UK; 3 Peninsula Medical School, The John Bull Building, Tamar Science Park, Plymouth, PL6 8BU, UK; 4 Institute of Evolutionary Biology, University of Edinburgh, King's Buildings, West Mains Road, Edinburgh, EH9 3JT, UK

## Abstract

**Background:**

Explaining public-goods cooperation is a challenge for evolutionary biology. However, cooperation is expected to more readily evolve if it imposes a smaller cost. Such costs of cooperation are expected to decline with increasing resource supply, an ecological parameter that varies widely in nature. We experimentally tested the effect of resource supply on the evolution of cooperation using two well-studied bacterial public-good traits: biofilm formation by *Pseudomonas fluorescens *and siderophore production by *Pseudomonas aeruginosa*.

**Results:**

The frequency of cooperative bacteria increased with resource supply in the context of both bacterial public-good traits. In both cases this was due to decreasing costs of investment into public-goods cooperation with increasing resource supply.

**Conclusion:**

Our empirical tests with bacteria suggest that public-goods cooperation is likely to increase with increasing resource supply due to reduced costs of cooperation, confirming that resource supply is an important factor in the evolution of cooperation.

## Background

Public-goods cooperation is widespread in nature but explaining this is a challenge for evolutionary biologists [1-3]. The central problem is that investment in the public-good is costly to individuals yet the public-good may be used by others, thus, all else being equal, cheats that reap the rewards of cooperation without making any investment should be able to invade a population of cooperators [2]. Kin selection provides a general solution to this social dilemma [3-5]: public-goods cooperation can be favoured if the benefits of cooperation are directed towards relatives with whom the cooperator shares genes. This is captured in Hamilton's rule which states that cooperation is favoured when *rb *> *c*, where *c *is the personal fitness cost for the actor, *b *is the fitness benefit to the recipient, and *r *is the genetic relatedness between the actor and recipient. Thus, provided that the indirect benefit (*rb*) accruing from cooperation exceeds the direct cost (*c*) of investment, then public-goods cooperation can evolve. While the importance of relatedness for the evolution of public-goods cooperation has been demonstrated in several recent empirical studies of microbes [6-8], the effect of variation in the cost of cooperation remains less well explored.

Resource supply is an important ecological variable that varies widely in nature and is known to have important evolutionary consequences [9]. Furthermore, the availability of resources is likely to mediate the individual cost of investing resources into public-goods cooperation [10]. For example, because investment of resource into growth ultimately gives diminishing returns, then the cost of diverting resources away from growth and into cooperative public goods production will be higher when resources are scarce and lower when resources are not limiting. Put another way, high resource supply is likely to reduce the relative cost of cooperation. This leads to the following testable prediction for binary cooperative traits (i.e., where individuals either cooperate or cheat): the fitness of cooperators and by extension the frequency of cooperators within the population should increase with increasing resource supply.

We experimentally tested this prediction using two bacterial cooperative traits. First, we considered biofilm formation in *Pseudomonas fluorescens *[11-14]. When propagated in spatially heterogeneous microcosms (a static glass vial containing nutrient-rich medium), populations of the ancestral smooth (SM) *P. fluorescens *genotype rapidly diversify, generating a range of niche specialist genotypes by mutation that are maintained by negative frequency dependent selection [15]. The wrinkly-spreader (WS) morph is ecologically dominant [16,17], forming a biofilm at the air-broth interface through constitutive overproduction of cellulosic polymer [18]. While over-expression of cellulosic polymer is individually costly (as demonstrated by the reduced exponential growth rate of WS relative to SM [11,19]), its production provides a group benefit to WS because colonisation of the air-broth interface niche allows improved access to oxygen, a limiting resource [11]. Clonal WS biofilms have been found to be susceptible to rapid invasion by SM genotypes that arise by mutation from WS over the course of several days [11-14]. In this context SM are cheats, gaining the benefit of inhabiting the air-broth interface while making no contribution to the integrity of the biofilm, which is significantly weaker in the presence of cheating SM genotypes [11]. SM may also defect from the biofilm entirely, inhabiting the less productive broth phase of the microcosm [11,12]. Broth dwelling bacteria are asocial with respect to biofilm formation, and appear to pay a considerable cost in the presence of well-developed WS biofilms (present after 2–3 days growth in rich media) that restrict diffusion of oxygen into the broth causing cell death [11]. Previous work suggests that the evolutionary emergence of WS via adaptive radiation from SM may be resource limited, but the impact of resources on cooperation and cheating was not explicitly investigated [20]. Four independent WS genotypes were experimentally evolved for 16-days at a range of carbohydrate and amino acid resource concentrations under conditions known to promote biofilm formation [12]. After 16 days, samples were first taken from the broth phase of cultures and plated onto agar. This allowed us to identify the frequency of bacteria that inhabited the broth, and hence were asocial with relation to biofilm formation. The remainder of the culture was then homogenised, to break down the biofilm, and a further sample taken and plated onto agar. This allowed us to determine the frequency of cooperators (i.e., biofilm-forming WS) and cheats (i.e., biofilm dwelling SM).

Second, we carried out experiments on siderophore production by the pathogenic bacterium, *Pseudomonas aeruginosa *[21]. Siderophores are extracellular iron-scavenging molecules that are facultatively produced in response to iron limitation [22]. Like biofilm formation, siderophore production is vulnerable to invasion by non-producing cheats, because siderophore production is metabolically costly but provides a benefit to local conspecifics with appropriate receptors [6,7,23,24]. We competed wild-type *P. aeruginosa *(cooperators) with cheats that were unable to produce the primary siderophore, pyoverdine, under iron-limited conditions and a range of carbohydrate and amino acid resource concentrations. After three days the populations were homogenised and plated onto agar and the proportions of cooperators and cheats counted. Fitness of cooperators was then calculated relative to cheats.

## Results and Discussion

### Biofilm cooperation

**Figure 1:**
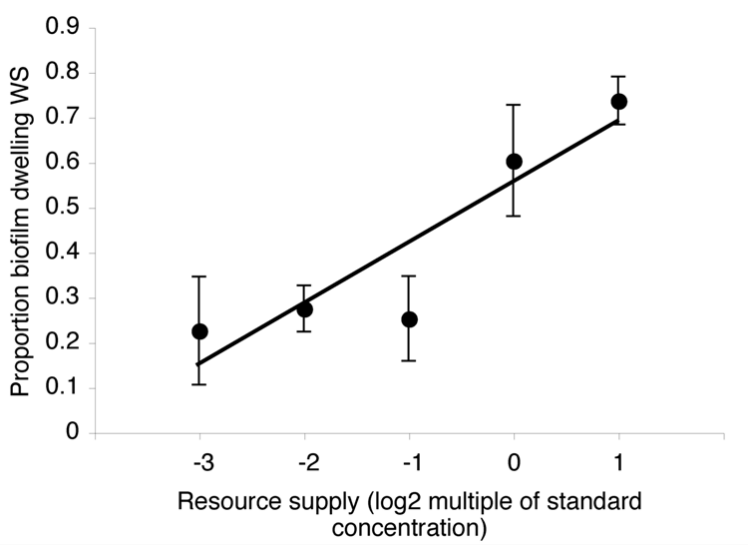
**Selection for cooperative biofilm formation increases with increasing resource supply**. Dots represent mean ± Standard Error (SE) proportion of biofilm dwelling WS (i.e., density WS/total density) on day 16 of the experiment. These data show that the proportion of cooperative WS increases with increasing resource supply.

Increasing resource supply increased total bacterial population density, demonstrating that the manipulated resources were indeed limiting and non-toxic (data not shown; founding genotype, *F*_3,15 _= 1.47, *P *= 0.2; log_2_(resource supply), *F*_1,15 _= 50.41, *P *< 0.0001). Crucially, biofilms formed under all treatments. To test whether selection for cooperation increased with increasing resource supply we calculated the proportion of biofilm-forming WS within each population (i.e., WS density/total density). Consistent with our predictions, the proportion of biofilm-forming WS increased with increasing resource supply (Figure 1; founding genotype, *F*_3,15 _= 1.01, *P *= 0.4; log_2_(resource supply), *F*_1,15 _= 24.26, *P *< 0.0001). This suggests that selection to cease cooperative investment into biofilm production (i.e., bacteria inhabiting the broth and SM cheats within the biofilm) decreased with increasing resource supply.

**Figure 2:**
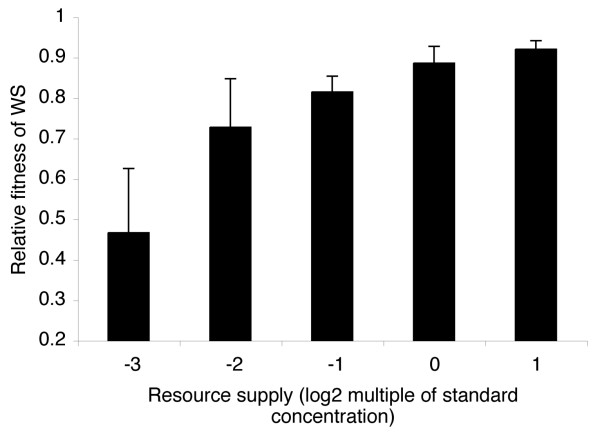
**The cost of cooperation decreases with increasing resource supply**. Bars represent mean + SE fitness of WS relative to SM after 24 hours of competition in shaken microcosms where the WS phenotype is purely costly due to the prevention of biofilm formation. These data show that the fitness of the cooperative WS phenotype relative to the SM phenotype increases with increasing resource supply.

Our verbal argument suggested that the mechanism underlying this pattern is decreasing physiological costs of cooperation with increasing resource supply. We tested this by growing WS and SM under conditions that do not allow biofilm formation such that the WS phenotype is purely costly (i.e., shaken tubes). In support of our predictions, the growth rate of WS relative to SM increased with increasing resource supply (Figure 2; WS genotype, *F*_3,15 _= 5.29, *P *= 0.01; log_2_(resource supply), *F*_1,15 _= 51.94, *P *< 0.0001). This confirms that increasing resource supply did indeed reduce the cost of the cooperative WS phenotype.

**Figure 3:**
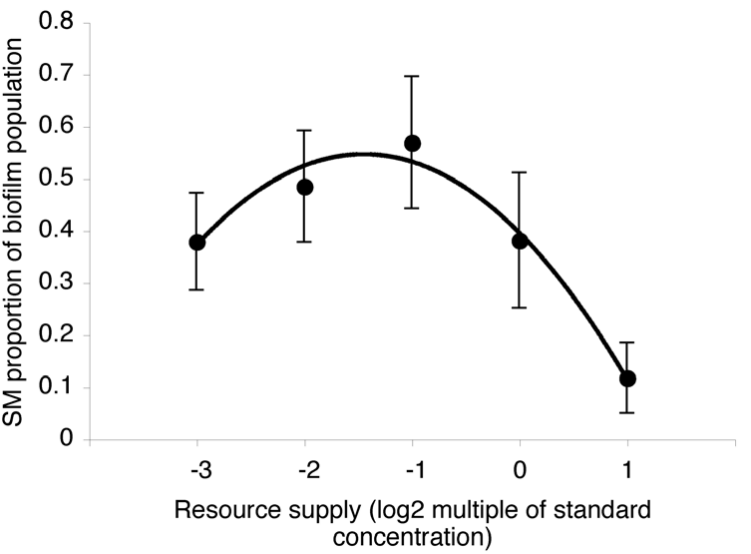
**Selection for biofilm dwelling cheats peaks at intermediate resource supply**. Dots represent mean ± SE proportion of biofilm dwelling population with SM colony morphology (i.e., biofilm SM density/[biofilm SM density + WS density]). These data show that selection for biofilm-dwelling SM, rather than SM *per se*, peaked at intermediate resource supply.

Resource supply may also affect the benefits of cooperation: variable benefits of biofilm dwelling across the resource supply gradient could have altered the strength of selection upon SM for inhabiting the biofilm relative to selection for leaving the biofilm entirely. As density increased with increasing resource supply, it is likely that competition for oxygen within the microcosm also increased. This is likely to increase selection for inhabiting the biofilm. In support of this, the proportion of the SM population inhabiting the biofilm as cheats compared to asocial broth dwelling (i.e., biofilm SM density/total SM density) increased linearly with resource supply (data not shown; founding genotype, *F*_3,15 _= 0.17, *P *= 0.9; log_2_(resource supply), *F*_1,15 _= 11.64, *P *= 0.004). However, opposing this, increasing resource supply reduced the physiological costs of cooperation (Figure 2), thus reducing the selective benefit of cheating relative to cooperating. These opposing selection pressures lead to the prediction of a unimodal relationship between resource supply and the proportion of cheating compared to cooperating within the biofilm. To test this we calculated the proportion of SM compared to WS within each biofilm (i.e., biofilm SM density/total biofilm density). In accordance with our verbal argument, the proportion of SM cheats peaked at intermediate resource supply (Figure 3; founding genotype, *F*_3,14 _= 2.27, *P *= 0.125; linear log_2_(resource supply), *F*_1,14 _= 20.37, *P *< 0.0001; quadratic log_2_(resource supply), *F*_1,14 _= 12.15, *P *= 0.004).

### Siderophore cooperation

**Figure 4:**
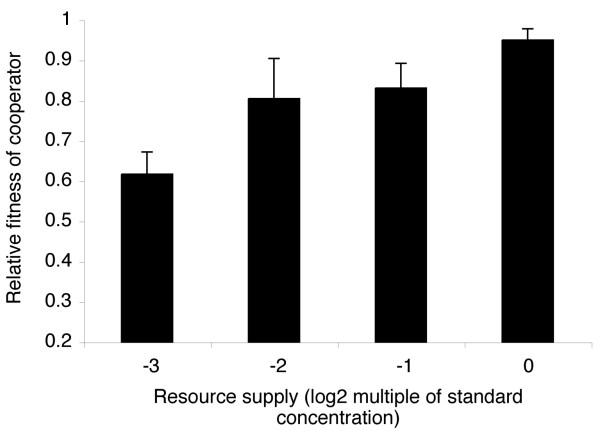
**The cost of siderophore cooperation decreases with increasing resource supply**. Bars represent mean ± SE fitness of siderophore producers relative to non-producers after 24 hours of competition. These data show that the fitness of cooperative siderophore producers relative to non-producing cheats increases with increasing resource supply.

As hypothesised, the fitness of cooperators relative to cheats increased with resource supply (Figure 4; *F*_1,10 _= 18.74, *P *= 0.001). This confirms our prediction that selection for cooperation increases with increasing resource supply. It should be noted that the fitness of cooperators did not exceed that of cheats under any resource supply conditions. This is because our experiment involves local (i.e., within population) competition: previous studies with this system have shown that global (i.e., between populations) competition is required for cooperators to have a net fitness advantage over cheats [6]. Because the media ingredients that were manipulated may have contained additional iron it is possible that increasing resource supply may have inadvertently increased iron availability if any additional iron could not be sufficiently chelated by apotransferrin. This may have resulted in down-regulation of siderophore production under high resource supply such that cooperators might have been fitter under high resources because they produce less public good. To test this, we grew pure cultures of cheats and cooperators along the resource gradient. Whereas pure cooperator and mixed cultures increased in density with increasing resource supply (*F*_1,10 _= 15, 132, respectively; *P *< 0.01, in both cases) there was no effect of resource supply on cheat density (*F*_1,10 _= 1.69, *P *> 0.2). This suggests that iron availability was not significantly altered across the resource supply gradient, and therefore that the cost of cooperation was indeed reduced through increased resource supply.

### Broader relevance

We have tested the effect on cooperation of the supply of resources that are not acquired directly through public-goods cooperation: a positive relationship was observed for two bacterial social traits. The crucial mechanism explaining the experimental results is simply that the cost of cooperation decreases with increasing resource supply. This arises because the benefit of investing resources into individual growth and reproduction is likely to show diminishing returns. Metabolic constraints (e.g., rate-yield trade-offs [25]) would suggest that this assumption is frequently, if not always, correct.

Situations where public-goods cooperation is either not directly involved in resource acquisition, or supply of the limited resource targeted by social action is not strongly correlated with the supply of other essential resources, are likely to be common in nature. A relevant example is iron availability to bacterial pathogens within host organisms, the supply of which is often more restricted than that of other resources due to active sequestering by the host [26]. However, the relationship between cooperation and resource supply is unlikely to be monotonic if the manipulated resource is itself acquired through public-goods cooperation. For example, if the impact of oxygen availability on biofilm formation was investigated, it is likely that biofilm cooperation would be reduced at high oxygen supply rates because biofilms would not be necessary to obtain oxygen. Thus the net effect of resource supply is likely to depend upon the covariance of different types of resource, both those targeted by the public-good and those that provide raw materials for its production.

In summary, we have shown that the success of cooperation is crucially dependent on resource supply rate. Our experiments suggest that cooperation increases with increasing resource supply rate, due to decreasing costs of cooperation. These results along with observations from termite societies, where cooperative behaviours were highest in colonies with abundant food [10], highlight that the role for resource supply in mediating the costs and benefits of kin-selected traits may apply very generally indeed.

## Conclusion

We have shown, using empirical tests with bacteria, that public-goods cooperation increases with increasing resource supply due to reduced costs of cooperation, confirming that resource supply is an important factor in the evolution of cooperation.

## Methods

### Isolating P. fluorescens WS genotypes

Four replicate microcosms (30 mL glass universal containing 6 mL of King's B nutrient media) were inoculated with *Pseudomonas fluorescens *SBW25 to a total of approximately 10^7 ^cells. These were statically incubated for 6 days at 28°C, after which time all populations were vortexed and an aliquot diluted and plated onto KB agar. A single wrinkly-spreader colony was then isolated from each population for further study and stored at -80°C in 20% glycerol.

### Resource supply selection experiment

Populations were initiated with 10^7 ^cells of one of the isolated WS genotypes grown for 18 h under shaken conditions. These were then propagated under one of the following resource supply regimes: 0.125×, 0.25×, 0.5×, 1× and 2× standard KB, generated by serial dilution of KB medium into M-9 salt solution. These resource supply rates were selected following pilot studies to ascertain that, under all levels of resource supply, biofilm formation was possible and media was non-toxic. 6 uL of each culture was transferred to a fresh microcosm every 4 days over a 16-day period. After 16 days the broth phase of each population was sampled, then populations were homogenised and sampled. Samples were then plated onto agar and the frequencies of WS and SM colonies counted.

### Biofilm fitness assays

WS genotypes and the SM ancestor were grown in KB for 18 hours in shaken conditions to attain the same physiological state. A microcosm at each resource supply level was inoculated with 10^7 ^cells of a 50:50 mixture of one of the WS genotypes and the SM ancestor. Mixtures were plated onto KB agar to determine WS and SM densities. Microcosms were then incubated for 24 hours under shaken conditions, and plated onto KB agar to determine WS and SM densities. The fitness of WS relative to SM was then calculated.

### Siderophore fitness assays

We inoculated approximately 10^6 ^cells of overnight cultures of PA01, a pyoverdin-negative mutant, PA6609, or 1:1 mixtures of the two into wells of 96-well microtitre plates containing 150 ul of media. Four resource supply rates were used: M9 salts containing 2.5, 1.25, 0.625 & 0.3125 g of Casamino acids (sigma). All media were supplemented with 100 mg/ml human apo-transferrin and 20 mM NAHCO_3 _to chelate iron. Three replicates were established for each strain-resource supply combination. Cultures were propagated for 72 h in a 37°C static incubator. At the start and end of the experiment, cultures were pleated onto KB agar and the frequency of cooperators (green colonies) and cheats (white colonies) elucidated, and relative fitness calculated. Final densities were estimated from optical densities (OD^600^).

### Statistical analysis

All proportion data were arcsin-square-root transformed, density data were log_10 _transformed and relative fitness data were cubed prior to analysis to conform with GLM assumptions of homogeneity of variance and normality of residuals. Data were then analysed in GLMs fitting founding genotype as a random factor and log_2 _multiple of standard resource concentration as a linear (and in certain cases quadratic) covariate. Relative fitness (*W*) of cooperators was calculated from the ratio of the estimated Malthusian parameters (*m*) of the cooperators:cheats, *m *= ln (N_f_/N_0_), where N_0 _is the starting density and N_f _the final density. For the siderophore experiment resource supply was fitted as a covariate in a GLM.

## Authors' contributions

Original concept and development of hypotheses MAB, AB, AG. Biofilm experiments MAB. Siderophore experiments AB, DR. Statistical analysis MAB, AB. Drafting of manuscript MAB, AB, AG.
